# A radon-thoron isotope pair as a reliable earthquake precursor

**DOI:** 10.1038/srep13084

**Published:** 2015-08-13

**Authors:** Yong Hwa Oh, Guebuem Kim

**Affiliations:** 1 School of Earth and Environmental Sciences/Research Institute of Oceanography, Seoul National University, Seoul 151-747, Republic of Korea

## Abstract

Abnormal increases in radon (^222^Rn, half-life = 3.82 days) activity have occasionally been observed in underground environments before major earthquakes. However, ^222^Rn alone could not be used to forecast earthquakes since it can also be increased due to diffusive inputs over its lifetime. Here, we show that a very short-lived isotope, thoron (^220^Rn, half-life = 55.6 s; mean life = 80 s), in a cave can record earthquake signals without interference from other environmental effects. We monitored ^220^Rn together with ^222^Rn in air of a limestone-cave in Korea for one year. Unusually large ^220^Rn peaks were observed only in February 2011, preceding the 2011 M9.0 Tohoku-Oki Earthquake, Japan, while large ^222^Rn peaks were observed in both February 2011 and the summer. Based on our analyses, we suggest that the anomalous peaks of ^222^Rn and ^220^Rn activities observed in February were precursory signals related to the Tohoku-Oki Earthquake. Thus, the ^220^Rn-^222^Rn combined isotope pair method can present new opportunities for earthquake forecasting if the technique is extensively employed in earthquake monitoring networks around the world.

Earthquake prediction remains one of the most challenging and important problems faced by the scientific communities. During the last few decades, potential earthquake precursors, including ^222^Rn, Cl^−^, SO_4_^2−^, and stable isotope ratios (δ^2^H and δ^18^O) have been monitored in numerous fault and volcanic zones for earthquake prediction^1^,^2^,^3^,^4^,^5^,^6^. Such precursor signals are anticipated by the formation of micro cracks in fault zones or by groundwater mixing due to crustal dilation^1^,^6^. Stresses that develop prior to an earthquake are thought to be responsible for the release and accumulation of certain constituents that may be useful as tracers or precursors of these tectonic forces. However, these potential precursors have not been extensively used to forecast tectonic or volcanic activities because abnormal increases of these components also occur due to other environmental processes, including changing meteorological conditions. Among the potential earthquake precursors, ^222^Rn in soil and groundwater has shown the highest sensitivity because of its radioactive nature and origin in the subsurface^7^,^8^,^9^,^10^,^11^,^12^. However, ^222^Rn still suffers from interferences by meteorological phenomena and tidal forces^10^,^13^,^14^.

In order to test the concept of a dual isotopic tracer, we monitored both ^222^Rn and ^220^Rn every hour in a limestone cave (Seongryu Cave, Korea) from May 18, 2010 to June 17, 2011 (see Methods). To our knowledge, this represents the first study to evaluate this ^222^Rn-^220^Rn isotope pair in an underground environment as a precursor of earthquakes. Seongryu cave, which is ~250 Ma in age, is located in Seonyu Mountain (elevation: 199 m) in the eastern part of Korea (Fig. 1). The cave is at 20 m above the mean sea level and is ~330 m in length, 1–13 m in height, and has an entrance of ~1 m^2^. The main cave contains many branches, including three lakes, of which the two located near the entrance are affected by an outside stream^15^. More detailed description of this cave is available in Oh and Kim^16^.

For the ^220^Rn measurements, the position of the air-inlet above the cave floor is critical because ^220^Rn decays almost immediately (~5 minutes for 97% decay) after emanation from the source. Since ^220^Rn in the soil air should be in equilibrium with its parent ^224^Ra (even in the skin layer) due to its short half-life, variations in the ^220^Rn activity of soil air, including that of the porewater in rocks and soils would not be useful. Therefore, the air-inlet must be positioned at a height where the inputs of ^220^Rn from general environmental processes are minimal, but also where large inputs from earthquakes are detectable. In addition, the monitoring location should be isolated from the outside air, which can also generate ^220^Rn anomalies due to wind-driven skin flow through rocks and soils. The atmosphere in the first 130 m of Seongryu Cave is influenced by the outside air, while the atmosphere in the inner cave is almost stagnant^15^. Thus, in this study we chose an air-inlet position 0.2 m above the cave floor and 180 m from the entrance (Fig. 1). At this position, the noise from general meteorological driving forces was minimal and the meteorological conditions were relatively constant for both ^220^Rn and ^222^Rn. The activity of ^220^Rn was not detectable when the air-let was positioned 1.5 m above the cave floor due to its short half-life (see Supplementary Fig. S1). The activities of ^220^Rn were higher at the entrance site than at the monitoring site due to the influence of wind-driven skin flow (see Supplementary Fig. S2). At the monitoring site, ^222^Rn was both significantly stable and enriched relative to the entrance site (see Supplementary Fig. S2). Furthermore, since the activity ratios of ^222^Rn to ^220^Rn are high in the normal natural environment, there is a possibility of counts from ^222^Rn spilling over to ^220^Rn (see Methods). Previous study^16^ has shown that without a correction for this spillover, one could observe erroneous positive correlations between ^222^Rn and ^220^Rn.

Over the monitoring period, ^222^Rn activity was on average higher during the summer (avg. 645 ± 194 Bq m^−3^) than during the winter (avg. 140 ± 172 Bq m^−3^) (Fig. 2a), which is typical of ^222^Rn observations in caves around the world^17^,^18^. This seasonal variation in ^222^Rn activity is known to be due to the difference in air ventilation intensity. In contrast, ^220^Rn activity was on average higher in the winter (avg. 9.7 ± 10.1 Bq m^−3^) than in the summer (avg. 1.2 ± 3.7 Bq m^−3^) (Fig. 2b). In particular, we noted that both ^222^Rn and ^220^Rn showed high peaks in February 2011 that are decoupled from the general seasonal patterns (Fig. 3). Outside weather parameters (temperature, relative humidity, and pressure) showed large variations (−13.8–35.2 ^°^C, 7.5–97.9%, and 990.9–1033.6 mbar, respectively) relative to the inside air (10.7–15.7 ^°^C, 94–99.9%, and 999.0–1034.8 mbar, respectively).

With the exception of the anomalous peaks (red circles in Fig. 3), the daily average of ^222^Rn activities showed a significant positive correlation (n = 234, r^2 ^= 0.66) with the daily average of temperature (density) difference (the inside temperature is subtracted from the outside temperature) (Fig. 3a), which is typical of cave air behavior^17^,^18^. In general, the ^222^Rn activities in the outside air are approximately two orders of magnitude lower than those in cave airs. Thus, the lower ^222^Rn activities in winter could be due to greater ventilation of the denser outside air. In contrast, during the summer, ^222^Rn is trapped inside the cave due to the atmospheric stratification. In addition to the density difference, in some regions, the land surface humidity affects the activity of ^222^Rn in the underground air because pore space can be affected by water vapor condensation^19^. As such, we observed a positive correlation (n = 396, r^2 ^= 0.57) between ^222^Rn activity and the relative humidity of the outside air. The temperature and relative humidity of the inside air remained fairly constant, and they correlated weakly with ^222^Rn activities (r^2 ^= 0.27 and r^2 ^= 0.32, respectively). Precipitation and outside pressure showed no significant correlations with ^222^Rn activity (r^2 ^< 0.1 and r^2 ^= 0.12, respectively). Thus, the variations in ^222^Rn activities in the cave seem to be predominantly affected by variations in the outside temperature and humidity.

In contrast to ^222^Rn, the daily average of ^220^Rn activities, except for anomalous peaks, showed a negative correlation (n = 234, r^2^ = 0.51) with temperature difference (Fig. 3b) and relative humidity (r^2 ^= 0.41). However, poor correlations were observed between the activities of ^220^Rn and outside pressure and precipitation (r^2 ^= 0.15 and r^2 ^< 0.1, respectively). ^220^Rn activities in the winter were higher than those in the summer, resulting from higher ventilation which results in rapid advection of the pore air (with ^220^Rn already in equilibrium with ^224^Ra) in the cave, in the cold season.

The significant ^222^Rn and ^220^Rn anomalies observed in February 2011 (Fig. 3a–c) cannot be explained by normal meteorological variations, including episodic precipitation events (Fig. 3d). Thus, we consider that these anomalies may have been precursors of the Tohoku-Oki Earthquake, which occurred approximately one month later (Fig. 2). A recent study^20^ showed that the Tohoku-Oki Earthquake was preceded by a series of small earthquakes that started on 13 February 2011. Our results showed that the ^222^Rn alone could not distinguish the February anomalies from the summer peaks (Fig. 3a), however, there are clear anomalous signals based on ^220^Rn alone or the combined ^222^Rn vs. ^220^Rn plots (Fig. 3b,c).

In general, carrier gases (CO_2_, CH_4_, Ar, and He) play a critical role in controlling the migration and transport of trace gases (e.g., ^222^Rn) towards the surface^21^,^22^. From our results, we assume that the degassing of carrier gases peaked on February 12, 2011, reduced continuously until March 1, 2011, and then almost stopped on the day of the Tohoku-Oki Earthquake. Many studies have reported that carrier gases anomalies occurred days-weeks before earthquakes^23^.

Based on our results, we present three points of evidence to indicate that the ^220^Rn-^222^Rn isotope pair may be an excellent precursor of earthquakes: (1) ^220^Rn peaks during the anomalous period were much higher than those during normal periods over the year. The observed anomalies cannot be explained by any normal environmental conditions during the monitoring period (Figs 2 and 3); (2) A positive correlation was observed between ^220^Rn and ^222^Rn during the anomalous period, perhaps due to the venting of carrier-gases (e.g., CO_2_) from the sub-surface. On the other hand, negative correlations were observed more generally (Fig. 3c); (3) The peak hours of the ^220^Rn and ^222^Rn anomalies were episodic and decoupled from normal diurnal patterns (see Supplementary Fig. S3). In general, ^222^Rn showed a diurnal fluctuation pattern, particularly in spring and fall when temperature differences between day and night were largest, although this pattern is not seen for the short-lived ^220^Rn.

Although the monitoring site in this study is ~1200 km distant from the epicenter of the Tohoku-Oki Earthquake, the earthquake impacted the Korean Peninsula in a number of ways. The Korean Peninsula is located on the Eurasian tectonic plate, which extends to Japan. As a result of the Tohoku-Oki Earthquake it was estimated to have moved eastward by 1.2–5.6 cm^24^. In addition, 46 out of 320 monitoring wells in Korea showed changes in water level, temperature, and electrical conductivity as a result of the earthquake^25^,^26^. Therefore, it is not surprising that the radon isotope anomalies observed in Seongryu Cave may represent precursors of this extremely large earthquake.

On the basis of our observations of ^222^Rn and ^220^Rn, we suggest that a network of ^222^Rn-^220^Rn monitoring stations should be constructed to further verify the potential of this method for forecasting the locations and strengths of pending earthquakes. In order to filter out other environmental forcing factors, meteorological parameters and potential carrier gases (CO_2_, CH_4_) should also be monitored. Most importantly, we have to carefully select suitable natural or artificial cave systems for these stations. We can easily develop more sensitive ^220^Rn monitoring systems, data transmission setups to remote laboratories, and institute a canary program to automatically detect potential earthquake signals.

## Methods

A detailed description of the RAD7 radon monitor is available in Burnett *et al*.^27^ and Lane-Smith *et al*.^28^. Briefly, the RAD7 uses a silicon alpha detector to determine the daughters of ^222^Rn and ^220^Rn, ^218^Po (t_1/2_ = 3.05 min; 6.00 MeV), ^214^Po (t_1/2 _= 164 μs; 7.67 MeV), and ^216^Po (t_1/2 _= 0.15 s; 6.78 MeV). The surface of the detector uses electrostatic attraction to capture Po^+^ ions using an electric potential of 2000 to 2500 volts, and the alpha detector counts ^218^Po, ^216^Po, and ^214^Po alpha decays. We used both ^214^Po and ^218^Po peaks for the ^222^Rn measurements. An air filter is used at the entrance of the RAD7 to prevent dust particles and charged ions from entering the radon chamber. The internal air pump of the RAD7 (flow rate: 1 L min^–1^) was activated for 1 minute every 5 minutes to reduce maintenance labor in the humid cave air. In order to maintain relative humidity of <10%, which is necessary for a constant detection efficiency of the RAD7, a desiccant column and a passive moisture exchanger (DRYSTIK, Durridge Co.) were coupled to the air path of the RAD7. During the measurement period, a new desiccant column was replaced every 3 or 4 weeks.

In order to obtain accurate ^220^Rn activity data in the presence of extremely high ^222^Rn levels, we corrected for the ‘spillover effect’ of ^222^Rn to ^220^Rn by using the method of Chanyotha *et al*.^29^. Briefly, we assume that the efficiency of ^220^Rn detection is a quarter of that for ^222^Rn to account for thoron decay sample in the intake system (volume of sample tube + drying unit) and internal cell of the RAD7. For the correction of ‘spillover effect’, the spill factor is assumed to be 0.015, which is an average value calibrated and measured by Durridge. The spillover from one of the radon channels (C: ^214^Po) into the thoron channel (B: ^216^Po) can be corrected by the analysis software (Capture) provided by Durridge. Due to a lack of a precise calibration for ^220^Rn, the activity data are presented in ‘arbitrary units’. While there is uncertainty in the calibration of absolute ^220^Rn activities, it does not affect the interpretation of our results since the same procedures and conditions were held constant during the measurement period. The atmospheric parameters (temperature, relative humidity, and pressure) in the cave were measured hourly using external sensors (MSR145, MSR electronics) and stored in a data logger. Outside weather parameters were obtained from the Korea Meteorological Administration (KMA).

## Additional Information

**How to cite this article**: Oh, Y. and Kim, G. A radon-thoron isotope pair as a reliable earthquake precursor. *Sci. Rep*. **5**, 13084; doi: 10.1038/srep13084 (2015).

## Supplementary Material

Supplementary Information

## Figures and Tables

**Figure 1:**
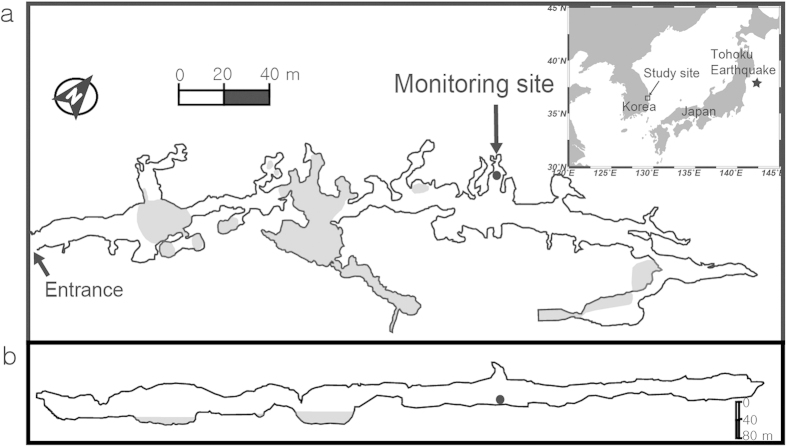
Location of the radon isotopes (^222^Rn and ^220^Rn) monitoring site in Seongryu Cave. (**a**) Location of the radon isotopes monitoring site and the layout of the cave. Grey areas denote lakes areas where the cave bottom is covered by water. (**b**) Vertical cross-section of the cave. This map was modified from Oh and Kim^16^.

**Figure 2:**
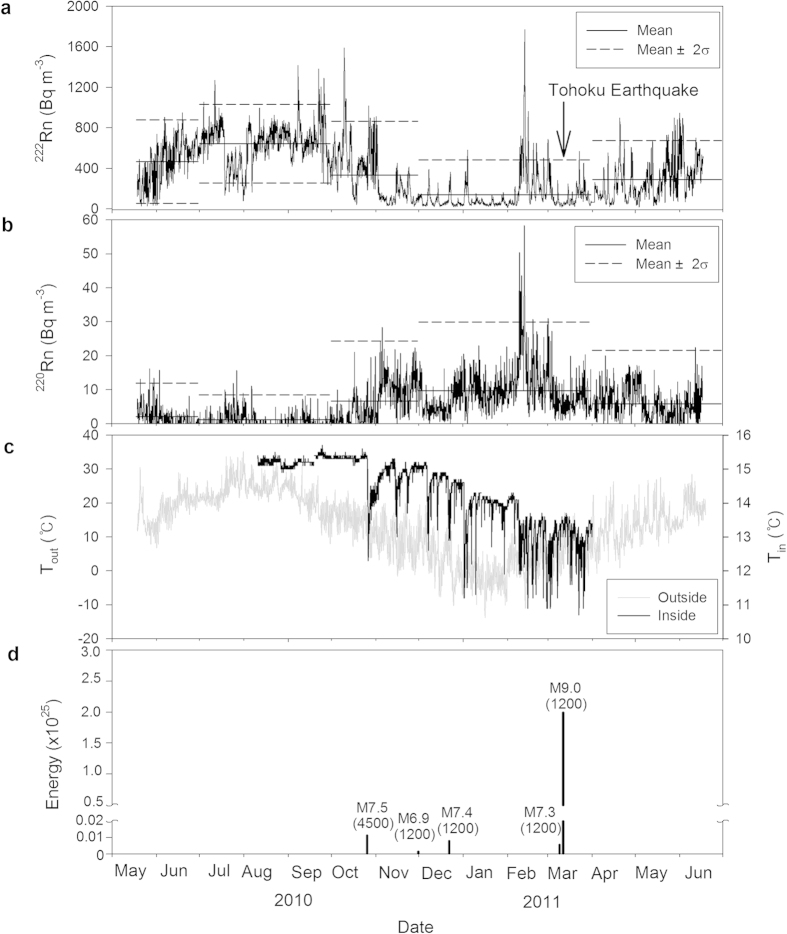
Variations in the activities of ^222^Rn and ^220^Rn in Seongryu Cave from May 2010 to June 2011. (**a)** Hourly variations in ^222^Rn activity. (**b**) Variations in 4-hour averaged ^220^Rn activity. (**c**) Variations in air temperature during the monitoring period, both inside and outside the cave. (**d**) Energy (unit: erg = 10^–7^ J) of earthquakes with magnitudes greater than M6.0 in Japan and Malaysia during the monitoring period. The seasonal (spring–winter) mean and mean ± 2σ (σ: standard deviation) values in (**a**) and (**b**) are shown for ^222^Rn and ^220^Rn activities. Numbers in parentheses in (**d**) denote distance (km) from the monitoring site. Due to a lack of a precise calibration for ^220^Rn, activities are presented in ‘arbitrary units’.

**Figure 3:**
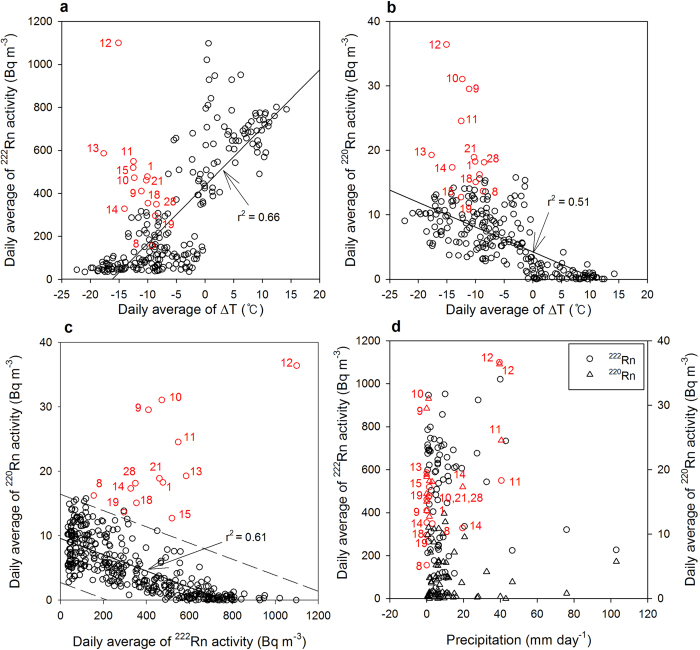
Relationships between the daily averages of ^222^Rn and ^220^Rn activities and weather parameters outside and inside the cave. Red circles denote the anomalous data above an upper limit of 99% prediction interval. Values denote the dates of the anomalous data in February 2011. Anomalous data were excluded from the correlation coefficients (r^2^). (**a**) Relationship between ^222^Rn activity and ΔT (temperature difference between the outside and inside of the cave). (**b)** Relationship between ^220^Rn activity and ΔT. (**c**) Relationship between the activities of ^222^Rn and ^220^Rn. The solid line denotes a regression line and the dashed lines denote the 99% prediction interval. (**d**) Relationship between the daily precipitation and activities of ^222^Rn and ^220^Rn.
