# Supplementary material for: A radon-thoron isotope pair as a reliable earthquake precursor
Source: Sci Rep. 2015 Aug 13;5:13084. doi: 10.1038/srep13084 (PMC4534786; doi:10.1038/srep13084)
Supplement: Supplementary Information [file srep13084-s1.pdf]

Supplementary Information

A radon-thoron isotope pair as a reliable earthquake precursor

Yong Hwa Oh and Guebuem Kim*

School of Earth and Environmental Sciences/Research Institute of Oceanography, Seoul National University, Seoul 151-747, Republic of Korea

*Correspondence to “gkim@snu.ac.kr”

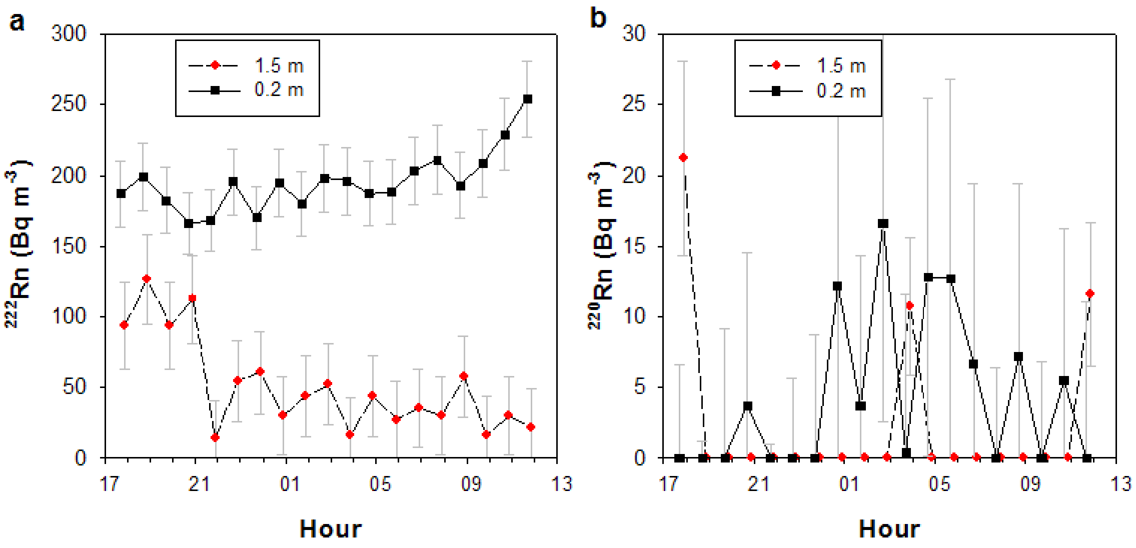

Supplementary Figure S1. Hourly variations in ^{222}Rn and ^{220}Rn activities at heights of 0.2 and 1.5 m above the cave floor at the monitoring site on May 5-6, 2011. (a) Variations in ^{222}Rn activity. **(b)** Variations in ^{220}Rn activity. The activities of ^{220}Rn approached the detection limit of the RAD7 at the depth of 1.5 m.

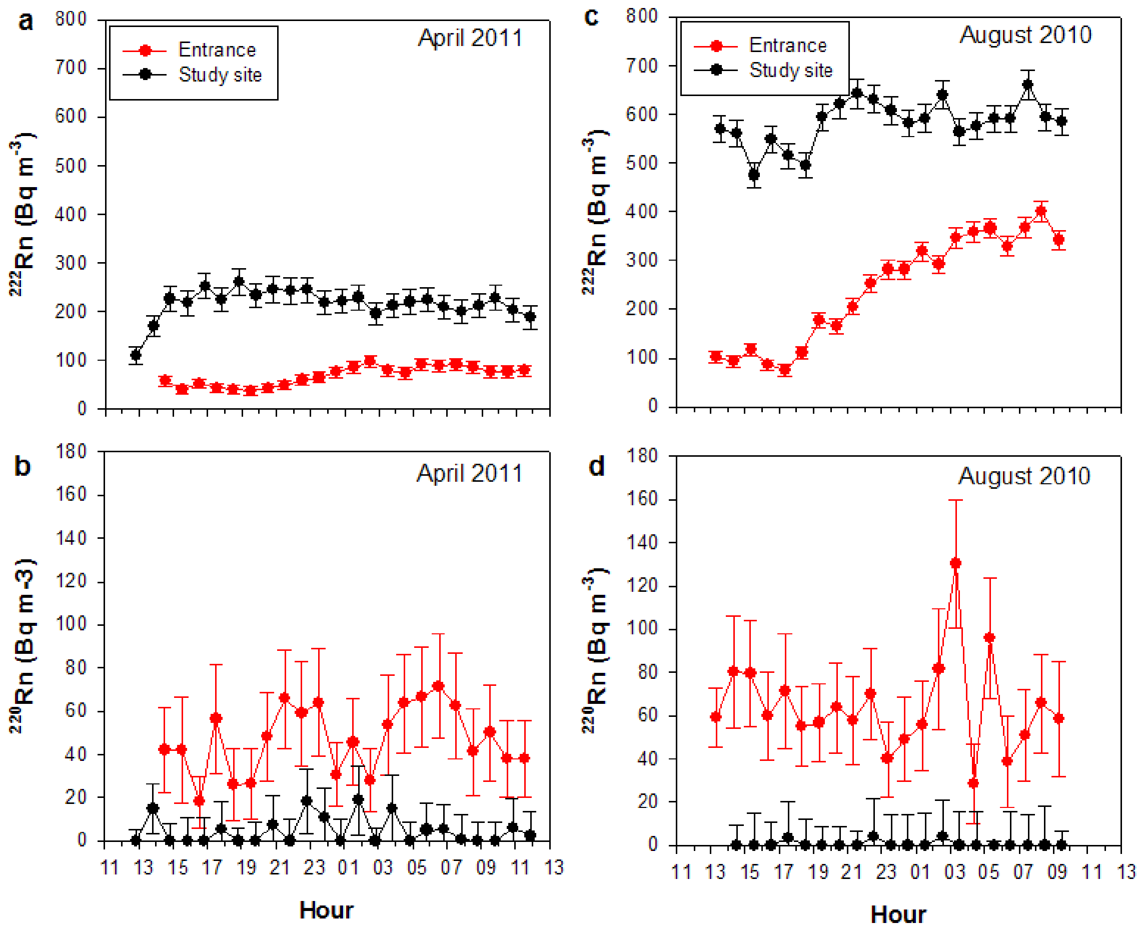

Supplementary Figure S2. Hourly variations in ^{222}Rn and ^{220}Rn activities at the entrance of the cave and the monitoring site inside the cave in two different seasons. **(a)** Variations in ^{222}Rn activity on April 2-3, 2011. **(b)** Variations in ^{220}Rn activity on April 2-3, 2011. **(c)** Variations in ^{222}Rn activity on August 9-10, 2010. **(d)** Variations in ^{220}Rn activity on August 9-10, 2010.

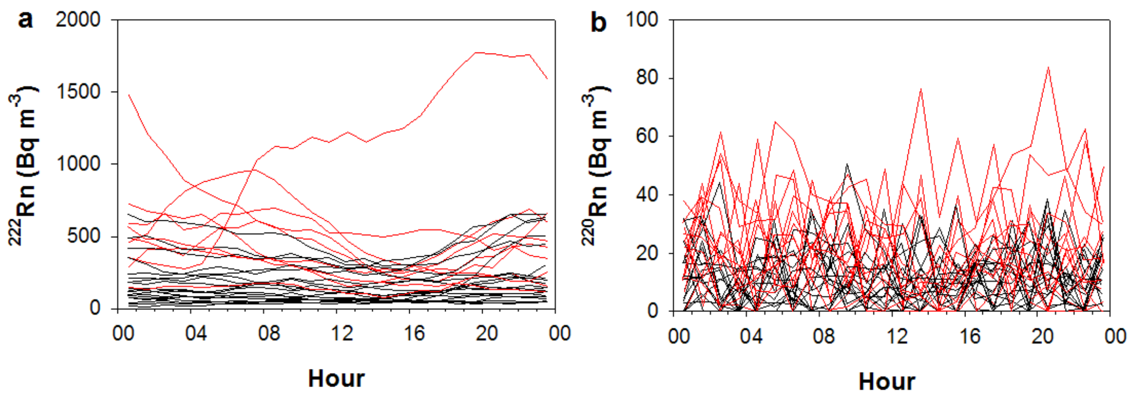

Supplementary Figure S3. Diurnal variations in ^{222}Rn and ^{220}Rn activities in February and March 2011. Red lines denote hourly variations in ^{222}Rn and ^{220}Rn activities for February 8-15, 18, 19, 21, 28 and March 1, 2011. **(a)** Variations in ^{222}Rn activity. **(b)** Variations in ^{220}Rn activity.
